# ‘Two-step’ percutaneous procedure for repairing the atrial septal defect with severe pulmonary hypertension: a case report

**DOI:** 10.1093/jscr/rjac612

**Published:** 2023-01-10

**Authors:** Liang Fu, Ruoxin Wang, Jinlong Zhao, Yinkai Ni, Zonghui Chen, Feng Li

**Affiliations:** Department of Cardiovascular Surgery, Shanghai Sixth People’s Hospital, Shanghai, People’s Republic of China; Department of Cardiovascular Surgery, Shanghai Sixth People’s Hospital, Shanghai, People’s Republic of China; Department of Cardiovascular Surgery, Shanghai Sixth People’s Hospital, Shanghai, People’s Republic of China; Department of Cardiovascular Surgery, Shanghai Sixth People’s Hospital, Shanghai, People’s Republic of China; Department of Cardiovascular Surgery, Shanghai Sixth People’s Hospital, Shanghai, People’s Republic of China; Department of Cardiovascular Surgery, Shanghai Sixth People’s Hospital, Shanghai, People’s Republic of China

## Abstract

Atrial septal defect is the most common type of congenital heart disease. Interventional closure is currently the best treatment for the atrial septal defect. However, the repair strategy for atrial septal defect accompanies by severe pulmonary hypertension remains controversial. We report a case of an atrial septal defect with severe pulmonary hypertension in which we applied a perforated occlude for atrial septal defect percutaneously at first. After 6 months, when the pulmonary pressure gradually went down, we completely blocked the reserved hole with a closure device percutaneously. We suggest that the stepwise procedure is an important treatment option for select patients with atrial septal defect accompanied by severe pulmonary hypertension.

## INTRODUCTION

The atrial septal defect is the most common congenital heart disease [1, 2]. Although considered to be a benign disease, it can still cause serious complications and fatalities with pathophysiological changes [3, 4], like severe pulmonary hypertension. The opinion of the closure of an atrial septal defect (ASD) with severe pulmonary hypertension (PH) is relatively controversial since the serious rise in pulmonary pressure after complete closure might be fatal [5]. It is critically important to determine whether the PH is reversible before the procedure. Takaya and colleagues reported that they chose the ‘treat-and-repair’ strategy, by using PAH-specific medication before complete ASD-closure, to treat the selective ASD-PH patients [6]. However, in that article, the estimated systolic pulmonary arterial pressure (PAP) is no higher than 78 mmHg, meanwhile the maximum ASD diameter was <23 mm [6]. Here we reported a patient, who had ASD (diameter 25 mm) accompany by severe PH (PAP 112 mmHg), successfully received our staging percutaneous closure procedures, and survived with an improvement in clinical outcomes.

## CASE REPORT

**Figure 1:**
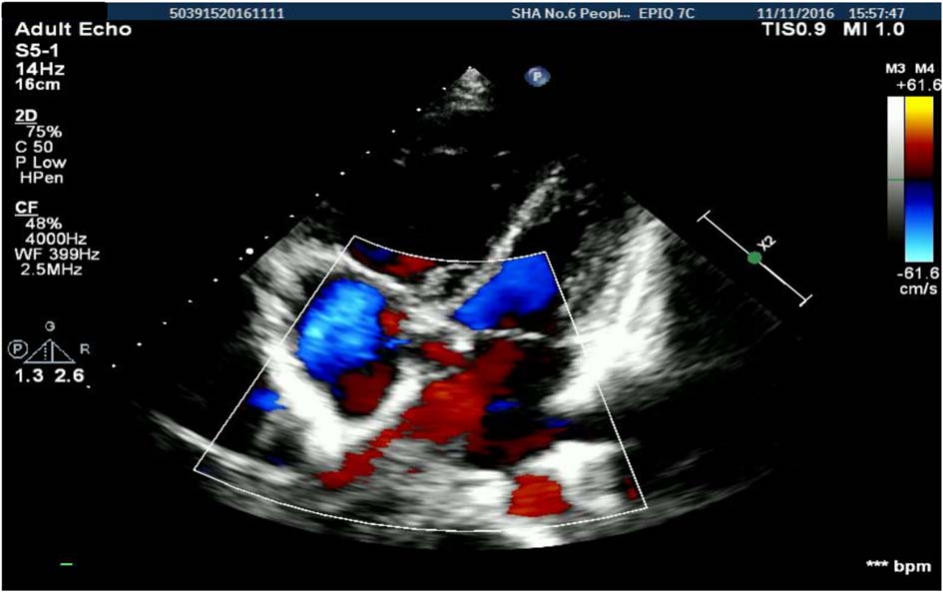
Transthoracic echocardiography suggested secondary atrial septal defect.

**Figure 2:**
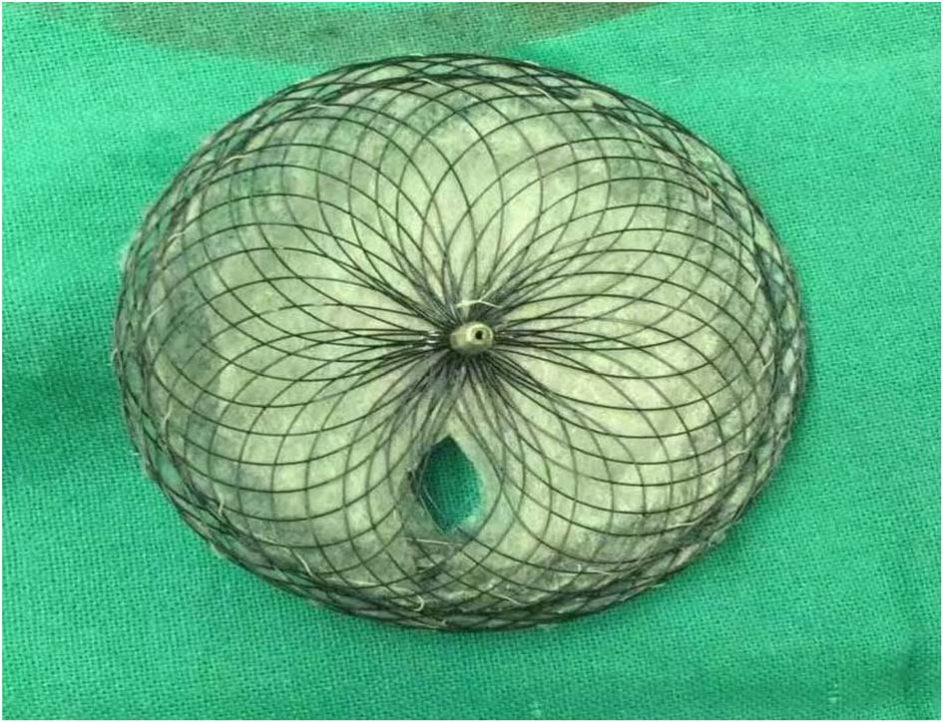
Occlusion of the occluder in advance reserved hole 5 mm.

**Figure 3:**
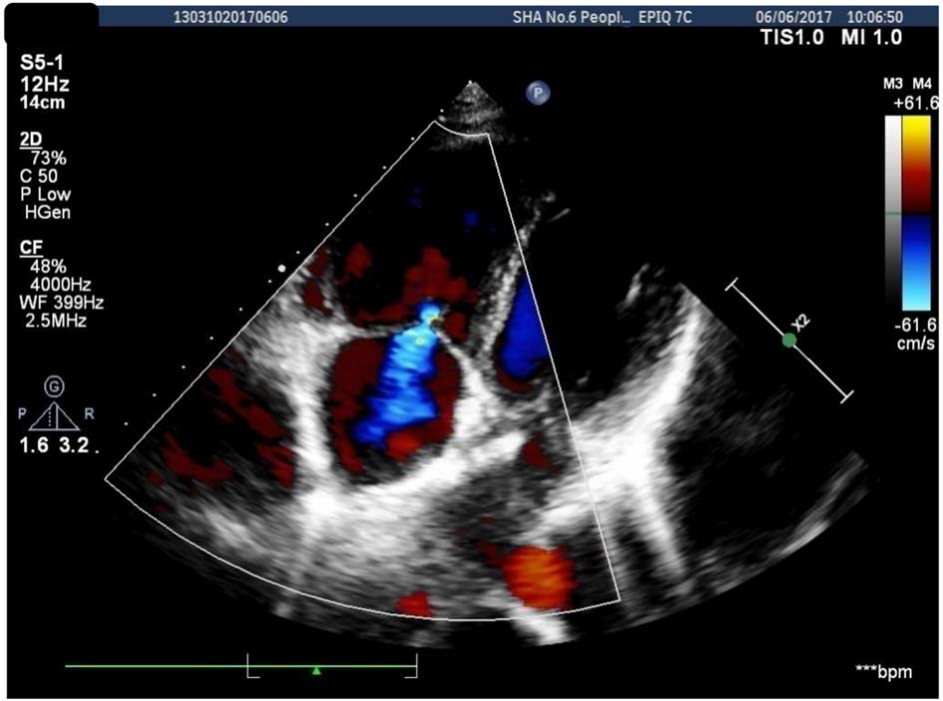
Re-plugged the 5-mm reserved hole.

A 26-years-old woman comes to the hospital because of a 1-week history of fatigue. She has no history of serious illness and cardiovascular examination history. She can do light to moderate physical activity without developing dyspnea. Vital signs are within normal limits. Cardiac auscultation shows a heart murmur at the second intercostal space on the left sternal border and a wide-split S2 that does not change with respiration. Physical examination shows no bluish-colored lips or digital clubbing. The transthoracic echocardiography indicated atrial septal defect (Type II, diameter 25 mm); left-to-right bidirectional shunt, severe pulmonary hypertension (estimate PAP: 114 mmHg); right atrium and ventricular dilatation with severe tricuspid regurgitation (Fig. 1). Blood gas analysis showed: PH: 7.36, PaO2: 77.2 mmHg and PaCO2: 36.1 mmHg. The electrocardiogram was normal. Chest radiograph demonstrated pulmonary artery segment bulging suggesting severe PH. Right-heart catheter verified severe PH (PAP: 112 mmHg). Therefore, repair or closure of the ASD would be a high risk of developing a probable surge of pulmonary artery pressure. After serious consideration, we decided to treat her with a manual fenestrated closure device. Under general anesthesia and transesophageal echocardiography (TEE) guidance, the patient has inserted a handmade fenestrated Amplatzer Septal Occluder (reserved hole 5 mm, Abbott, St. Paul, MN, USA; Fig 2) through the right femoral vein, occlusion of the occluder in advance. After the device was released, TEE showed a slight left-to-right shunt. After the procedure, the patient’s oxygen saturation was 100%. On the second day after surgery, this patient was discharged. Echocardiography was performed in the second month and sixth month since discharge, and the PAP was 68 and 34 mmHg, respectively. Meanwhile, tricuspid regurgitation was reduced to mild. The right atrium and right ventricle size also decreased compared with preoperative. Therefore, the patient’s PH was reversible since the PAP decreased significantly after the first-step procedure. Thus, after careful evaluation, we take the second stage of the procedure to re-plug the 5-mm reserved hole by using the Amplatzer Septal Occluder (Abbott, St. Paul, MN) to eliminate the shunt Fig. 3). The surgery was successful and the patient was discharged the next day.

## DISCUSSION

Atrial septal defects (ASDs) are one of the most prevalent congenital cardiac anomalies in adults [1, 2]. Because of the high blood shunt from left-to-right, ASDs can cause several changes in the cardiovascular system [7]: right-heart-chambers enlargement, tricuspid regurgitation, myocardial cell hypertrophy and cellular injury, right ventricular remodeling, most significantly, the increasing preload of the right heart can cause pulmonary over circulation [8]. Symptomatic benefits and improved quality of life are observed in all age groups after ASDs closure [6, 9]. Even PH is a relatively rare complication even amongst large ASDs [10]. Nevertheless, the risk of PH complicating ASD increases with advanced age [11, 12]. However, several articles report sudden death [13] or a relatively worse prognosis after late ASD-closure in ASD-PH patients [5, 14, 15]. Hence the repair strategy for ASD-PH patients is controversial.

Here, in this case, we performed the ‘two-step’ procedure to repair the ASD with severe PH percutaneously. We occluded the ASD with a handmade fenestrated closure device percutaneously at first. Postoperative follow-up confirmed that the PAP decreased significantly with tricuspid regurgitation reduced. After 6 months, we completely blocked the reserved hole with a closure device percutaneously. Further experience and large samples of clinical data are still needed for further study and validation, but for now, we suggest that the stepwise procedure is an important treatment option for select patients with ASD-PH. In addition, the follow-up evaluation to determine whether the reserved holes be re-plugged if necessary.
